# Oncogenic Role of PVT1 and Therapeutic Implications

**DOI:** 10.3389/fonc.2020.00017

**Published:** 2020-02-04

**Authors:** Onayemi Titilayo Onagoruwa, Gargi Pal, Chika Ochu, Olorunseun O. Ogunwobi

**Affiliations:** Hunter College (CUNY), New York, NY, United States

**Keywords:** PVT1, oncogene, therapeutic target, cell cycle, molecular signaling

## Abstract

PVT1, a long non-coding RNA has been implicated in a variety of human cancers. Recent advancements have led to increasing discovery of the critical roles of PVT1 in cancer initiation and progression. Novel insight is emerging about PVT1's mechanism of action in different cancers. Identifying and understanding the variety of activities of PVT1 involved in cancers is a necessity for the development of PVT1 as a diagnostic biomarker or therapeutic target in cancers where PVT1 is dysregulated. PVT1's varied activities include overexpression, modulation of miRNA expression, protein interactions, targeting of regulatory genes, formation of fusion genes, functioning as a competing endogenous RNA (ceRNA), and interactions with MYC, among many others. Furthermore, bioinformatic analysis of PVT1 interactions in cancers has aided understanding of the numerous pathways involved in PVT1 contribution to carcinogenesis in a cancer type—specific manner. However, these recent findings show that there is much more to be learned to be able to fully exploit PVT1 for cancer prognostication and therapy. In this review, we summarize some of the latest findings on PVT1's oncogenic activities, signaling networks and how targeting these networks can be a strategy for cancer therapy.

## Introduction

Plasmacytoma variant translocation 1 (PVT1), a long non-coding RNA encoded by the human PVT1 gene, is located in the well-known cancer-related region, 8q24. Non-coding RNAs (ncRNAs) are RNA transcripts from genes not encoding for a protein (1). Non-coding RNAs can be grouped into two major classes based on the transcript size: (1) small ncRNAs <200 bp, such as piRNAs (Piwi-associated RNAs), miRNAs (microRNAs), and snoRNAs (small nucleolar RNAs), and (2) long ncRNAs (lncRNAs), >200 bp (1, 2). LncRNAs are generated from the exonic and intronic regions of coding genes with the majority of them produced from the intergenic regions (3). Since most lncRNAs have a deficit of sequence conservation compared with the protein coding RNAs, they were initially thought to be of limited biological functions (4, 5). But several studies have demonstrated that the lncRNAs are involved in the regulation of biological processes involving regulation of gene expression, transcription, translation, cell cycle control, chromatin modification, and cellular differentiation (6–9). Aberrant expression of lncRNAs have been observed in cancer and may serve as predicting tools for patient outcomes (10, 11).

**Figure 1:**
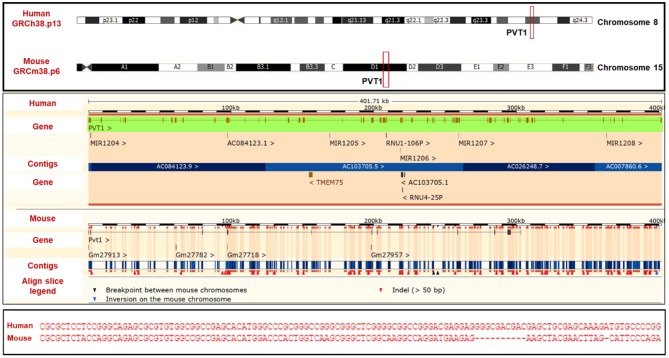
PVT1 **(Top)** PVT1 gene locus **(Middle)** alignment of human PVT1 and mouse pvt1 **(Bottom)** Sequence homology of human PVT1 and mouse pvt1. Figure was created using information from Ensembl genome browser.

The human PVT1 gene originates from an intergenic region on chromosome 8 (8q24) and it is homologous to the Pvt1 gene in mouse (chromosome 15) and rat (chromosome 7) (12, 13). Compared to the majority of lncRNAs, the PVT1 gene sequence is well-conserved across species (Figure 1). The oncogenic effects of PVT1 have been demonstrated by studies confirming its amplification/overexpression in many cancers (14–16). Lots of questions still need to be answered regarding the functions of PVT1, though strong suggestions point to its role as a regulatory RNA (13). Several studies have also suggested that PVT1 functions in a cell-type/ tissue-type specific manner (17–24). Currently, there is no one precise PVT1 mechanism of action that is unique to all the cancers, although there is frequently cancer progression when PVT1 is upregulated (8–10, 13, 15, 16).

Multiple studies carried out in cancer regulation have shown that miRNAs might be “sponged” by PVT1 [to reduce the miRNAs available to target the biological activity of mRNAs (22, 23, 25–27)]. PVT1 can also epigenetically silence miRNAs as part of its regulatory network in some cancers (28, 29). Transcription factors that play important roles during embryonic development, cell growth and cell proliferation are also major participants in tumorigenesis. Upregulation of transcription factors which act as activators in signal transduction is a major contribution of PVT1 to the regulation of carcinogenesis (30–32).

Many authors have also described the regulation of PVT1 in cancer by genes that suppress tumor growth (20, 33). Myc, a well-known oncogene situated in close proximity to PVT1 on chromosome 8 also functions as a PVT1 regulator. The human PVT1 gene contains two non-canonical MYC-binding sites (E-box CACGCG) in the promoter region proximal to the transcriptional start site (15). Other studies carried out on PVT1-Myc interactions have demonstrated established regulatory networks between these two (19, 34).

Certainly, identifying the molecular mechanisms of PVT1 could have important implications for therapeutically targeting cancer. Some factors are highly expressed in some cancers while they might be rarely expressed in some other forms of cancer. Thus, direct and specific approaches can be used to facilitate targeted anti-PVT1 therapies in patients with cancers. This article discusses the regulatory effects of PVT1 on cellular functions, other regulatory mechanisms including transcription factors and other signaling mechanisms. And we discuss the potential for exploiting these PVT1 regulated mechanisms for therapeutically targeting cancers.

## PVT1 Regulation of miRNAs

**Figure 2:**
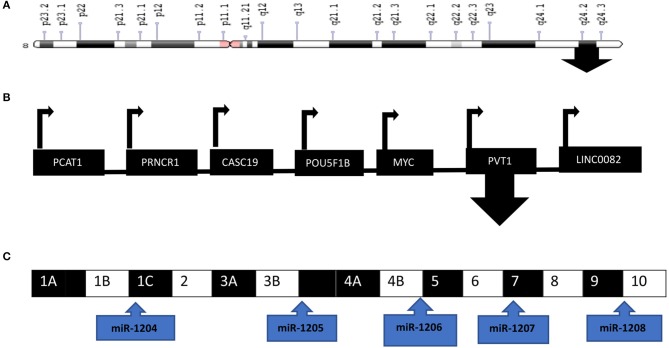
PVT1 is located at the 8q24 chromosomal region downstream of MYC. **(A)** Ideogram of chromosome 8 with the arrow located at 8q24. **(B)** PVT1 is one of the genes located at the 8q24 chromosomal region **(C)** PVT1 exons and the encoded miRNAs are depicted in the diagram. Illustration created with information from NCBI.

MicroRNAs (miRNAs) are 19-24 nucleotide non-coding RNA molecules that regulate the expression of target mRNAs both at the transcriptional and translational level (35, 36). PVT1 encodes six miRNAs, that is miR-1204, miR-1205, miR-1206, miR-1207-3p, miR-1207-5p, and miR-1208 (Figure 2). PVT1 and miR-1204 have a strong correlation of expression as they share promoters and are probably under the same regulatory circuit (37, 38). PVT1 acts as a competing endogenous RNA (ceRNA) to sponge i.e., regulate miRNA. PVT1 can bind to miRNA, thereby hindering the activity of miRNA on its target genes. However, this sponging mechanism is annulled in cancer.

In gastric cancer, miR-186 which binds directly to PVT1 exon 2 through complementary base coupling reduced the expression of the tumorigenic marker HIF-1a (25). miR-186-5p acts similarly to upregulate yes-associated protein 1 (YAP1) expression, thus promoting the tumorigenesis of hepatocellular carcinoma (26). Another miRNA, miR-152 was found to be negatively correlated with PVT1 in gastric cancer tissues (39). A luciferase assay verified the regulation of PVT1 by miR-152 via CD151 and FGF2 expression.

This mechanism of sponging by PVT1 was further observed in esophageal squamous cell carcinoma where PVT1 acted as a molecular sponge of miR-203 and LASP1. Down-regulation of LASP1 because of PVT1 inhibition was countered by transfection of miR-203 inhibitor or ectopic expression of LASP1 lacking the 3'-UTR (40). This appears to be a common trend in the regulation of PVT1 by miRNA as many other studies have confirmed. Tumor xenografts experiments suggested that silencing PVT1 sensitized NSCLC (Non-small-cell lung carcinoma) cells to radiotherapy *in vivo*, and this could be reverted by miR-195 inhibitor (41). An understanding of the level at which sponging by PVT1 take place was confirmed by measuring the effect of PVT1 on pre-miR-448 and pri-miR-448 in pancreatic cancer cells (42). PVT1 could not affect the level of pre-miR-448 and pri-miR-448 and this points toward an evidence of post-transcriptional regulation.

While most studies have indicated only one miRNA being targeted by PVT1 in most cancers, this should not be taken as the norm. More than one miRNA can be targeted by PVT1 in a cancer. In glioma cells, miR-190a-5p and miR-488-3p were both downregulated by PVT1 (43). And studies done on osteosarcoma separately have found different miRNAs as PVT1 targets (27, 44). A negative correlation dominates the relationship between PVT1 and miRNAs in cancer. This looks like a regular and predictable trend for most cancers. On the contrary, a positive correlation in expression was observed between PVT1 and miR-1207-5p in breast cancer cells, with the overexpression of both promoting cell proliferation of breast cancer cells. miR-1207-5p is produced by PVT1 introns (37).

## Epigenetic Regulation by PVT1

Epigenetic studies provide additional details about the interaction of PVT1 and miRNAs. PVT1 is one of the IncRNAs identified in association with PRC2 to regulate gene expression. Studies show that the upregulation of PVT1 favors its binding to EZH2 (Enhancer of Zeste Homolog-2), a subunit of Polycomb Repressive Complex 2 (PRC2) to inhibit miR-200b expression by increasing histone H3 lysine 27 trimethylation (H3K27me2) on the miR-200b promoter in cervical cancer (28). This demonstrates epigenetic silencing of a miRNA by PVT1. A classification of Diffuse Intrinsic Pontine Glioma (DIPG) in the pediatric group based on whole genome sequencing, analysis of copy number variations, expression and CpG island methylation led to the identification of subgroups including (H3-K27M, Silent, MYCN) in addition to mutation in ACVR1 (activin receptor) in 20% of DIPGs examined (45). A number of PVT1/MYC loci gains/amplifications discovered among the DIPGs occurred in the H3-K27M subgroup (histone 3 mutation). This H3-K27M subgroup demonstrated frequent genome instability as well as TP53 mutations and telomere length instability suggesting the need for multiple targeting of molecular abnormalities in this mostly fatal pediatric cancer.

A significant decrease in thyroid-stimulating hormone receptor (TSHR), a major regulator of thyroid metabolism, was confirmed by RNA immunoprecipitation (RIP) assay when PVT1 was silenced (21). This effect was modulated by PVT1 binding to EZH2 in thyroid cells. Consistently, the transcription of LATS2 which is suggested to be a tumor suppressor gene that is dysregulated in NSCLC (Non-small-cell lung carcinoma), was repressed by the recruitment of EZH2 by PVT1 (46). Many studies have suggested that there is a positive correlation between the expression level of EZH2 and PVT1 in many types of cancer. *In vitro* and *in vivo* studies verified that PVT1 overexpression could up-regulate EZH2 mRNA and protein levels in glioma (47).

However, protein–RNA immunoprecipitation assays confirmed that *PVT1* directly bind FOXM1 protein in gastric cancer cells with no significant change in the protein level of EZH2 (48). This could be due to PVT1 tissue specific mechanism of action. No study has been carried up to date out to compare how PVT1 modulates EZH2 in different cells.

## PVT1: A Regulator of the Cell Cycle

In a parallel or as an alternative mechanism, PVT1 also modulates cell cycle regulatory proteins [cyclin-dependent kinase (CDK) proteins and cyclins]. PVT1 negatively regulates the expression of p15 and p16 and its inhibition may contribute to cell-cycle arrest in gastric cancer (20). In thyroid cancer cells, data revealed that PVT1 suppression arrested the cell cycle at G0/G1 stage and decreased cyclin D1 expression (21). This effect appears to be mediated in conjunction with miRNA, and not PVT1 acting solely to regulate the cell cycle in this interaction. For example, in breast cancer cells, the percentage of breast cancer cells at G2 phase increased after transfection with the PVT1-derived miR-1207-5p mimic compared with the control (49). In another study by Chen et al. silenced PVT1 decreased the relative expression level of cyclin D1 and miR200c by binding to EZH2 (50).

The cyclin-dependent kinase (CDK) inhibitor p21 promotes cell cycle arrest by inhibiting CDK2 and CDK1 activity (51). An assessment of the effect of PVT1 on p21 expression in PANC-1 cells revealed a significantly increase in p21 at the transcriptional level when PVT1 was suppressed (33). Additionally, the tumor promoting activity of PVT1 was confirmed to be partially dependent on the negatively regulation on p21 in breast cancer (52).

A pathway analysis of the upregulated genes in the PVT1-overexpressing hepatocellular carcinoma cells revealed that the main pathway associated with PVT1 overexpression was the cell cycle pathway (5). Upregulated cell cycle genes in the PVT1 overexpressed cells were detected by microarray and confirmed by western blotting. Further studies on the cell cycle regulation by PVT1 in cancer are required for better comprehension on its function.

## PVT1 Mediates Drug Resistance

Drug resistance poses a great challenge to cancer treatment. It is a major determinant of patient mortality. Identifying and understanding the molecular processes of PVT1 impact on drug resistance will allow for more precise therapeutic interventions in cancer. The role of PVT1 in cisplatin resistance gastric cancer was explored by examining the effects of PVT1 on the expression of some genes associated with drug resistance. qRT-PCR and western blotting studies revealed that PVT1 up-regulation increased the expression of MDR1, MRP, mammalian target of rapamycin (mTOR), and hypoxia inducible factor-1 (HIF-1a) (53).

An increase in the expression of EZH2 has been identified as a key role in cancer progression and drug resistance (54). PVT1 has been identified as one of the LncRNAs that recruit EZH2 to consolidate their oncogenic roles (21). A study done on gemcitabine resistance in pancreatic cancer noted that curcumin down-regulates the expression of EZH2, PVT1 and their down-stream targets in gemcitabine-resistant cells (55). The role of PVT1 in mediating radiation resistance, though not as extensively studied compared with drug resistance, has shown that there are numerous pathways responsible for drug resistance in cancer. PVT1 fostered radiotherapy resistance in nasopharyngeal carcinoma by downregulating cleaved caspase-9, cleaved caspase-7, and cleaved PARP, thereby inhibiting apoptosis and subsequently causing radiation resistance (56). Altogether, PVT1 has been shown to be a promising target for treating drug resistance in cancer therapy.

## PVT1 Is a Participant in Multiple Signaling Pathways

Numerous pathways have been linked to PVT1. This is certain for a lncRNA that has been implicated in almost every type of cancer known. See Table 1 for a list of biological axes and signaling pathways linked to PVT1 in cancer.

**Table 1:** Signaling pathways linked to PVT1 in cancer.

**Pathway/axis**	**Type of cancer**	**PVT1 activity**	**References**
TGF-β signaling	Colorectal cancer	*PVT-1* knockdown promoted apoptosis via TGF-β signaling activation in CRC cells	(57)
ATM/Chk2/p53 signaling	Nasopharyngeal carcinoma	PVT1 can promote DNA repair by phosphorylation of ATM/Chk2/p53 signaling pathway	(44)
KLF5/beta-catenin signaling	Triple-negative breast cancer (TNBC)	PVT1 promotes proliferation and tumorigenesis in TNBC through stabilizing KLF5 and promoting CTNNB1 expression	(14)
STAT3/VEGFA signaling	Gastric cancer	PVT1 enhances the activation of STAT3 signaling pathway, thereby increasing VEGFA expression to induce angiogenesis	(58)
PRC2-PVT1-c-Myc axis	Pancreatic cancer	PRC2-PVT1-c-Myc is inhibited by curcumin, which enhances sensitivity of cancer cells to chemotherapeutic agents by targeting cancer stem cells	(43)
TGF-β/Smad signaling	Pancreatic cancer	PVT1 promotes epithelial-mesenchymal transition	(59)
miR-30d-5p/RUNX2 axis	Colon cancer	PVT1 could promote metastasis and proliferation of colon cancer	(15)
PI3K/AKT signaling	Nasopharyngeal carcinoma	Pvt1 promotes cancer stem cell–like properties in NPC cells by inhibiting miR-1207 and activating the PI3K/AKT signal pathway	(60)
PVT1/miR-203/LASP1 axis	Esophageal cancer	PVT1 could inhibit esophageal squamous cell carcinoma cell proliferation, migration and tumorigenesis. PVT1 could act as a molecular sponge of miR-203 and LASP1	(22)
EGFR signaling	Clear cell renal cell carcinoma (ccRCC)	PVT1 activated the EGFR pathway and participated in the progression of ccRCC	(61)
PVT1-miR-190a-5p/miR-488-3p-MEF2C-JAGGED1 axis	Glioma	Silencing PVT1 inhibited the malignant behaviors of glioma via up-regulating miR-190a-5p or miR-488-3p. miR-190a-5p or miR-488-3p suppressed oncogenesis through regulating oncogene JAGGED1, which was mediated by transcriptional factor MEF2C	(29)
PVT1/Mcl-1 axis	Clear cell renal cell carcinoma	PVT1 increased Mcl-1 (an antiapoptotic factor and regulator of apoptosis) mRNA levels in renal cancer cells by promoting its stability	(50)

## PVT1 and MYC in Cancer

PVT1 and c-Myc are both located on the 8q24 chromosomal locus (a frequent site for copy number gain and alterations in cancer) (62). The human PVT-1 gene contains two non-canonical MYC-binding sites (E-box CACGCG) in the promoter region proximal to the transcriptional start site (15). In a study by Carramusa et al., it was indicated that one of the two E-boxes is important for PVT1 promoter transcriptional regulation by c-Myc proteins (27). ChIP analysis of the same region in neuroblastoma cells revealed a novel network between PVT1 and N-Myc.

Many studies have long established the co-amplification of c-Myc and PVT1 in many cancers (57, 63–66). The upregulation of the expression of both PVT1 and c-Myc has been implicated and confirmed by observing a corresponding decrease in the level of c-myc upon silencing of PVT1. PVT1 has been shown to be an activator of c-Myc transcription, and likewise, c-Myc has also been implicated as a PVT1 activator in cancer cells (12). The possibility of common functional pathways has been described, yet, there is also evidence for independent contribution of PVT1 and MYC to tumorigenesis.

By utilizing chromosome engineering in transgenic mice models, it was demonstrated that a combination of PVT1 and MYC is essential to the formation of tumors (6). Extra copies of the MYC gene along with extra copies of Pvt1, Ccdc26, and Gsdmc (all located in the 8q24.21 chromosomal region) were shown to be necessary for the formation of mammary tumors. Alongside in the same study, PVT1 RNA and the MYC protein were shown to be significantly associated in primary tumors while siPVT1 resulted in repression of MYC protein level.

One of the proposed mechanisms of action for this correlation between PVT1 and MYC include PVT1 regulating and stabilizing the MYC protein by preventing MYC from proteolytic degradation. As mentioned earlier in this review, copy number changes found in the pediatric DIPG subgroup K27M-H3.3 included gains/amplifications of the MYC/PVT1 loci (45). The study suggested that the amplified MYC/PVT1 locus was as a result of K27M-H3.3 mutations.

Another proposition for the PVT1 and MYC linkage is the activation of regulators by the interaction of the increase in copy number of PVT1 and MYC to induce tumorigenesis. An example of such regulators is RSPO1. Elevated levels of RSPO1 (an activator of the canonical Wnt signaling pathway with essential roles in ovary determination) combined with PVT1 and MYC transcripts were found in human breast cancer tumors than tumors with low levels of MYC (67). This reciprocal action might involve other mediators which are unknown presently.

Even though the amplification of 8q24 has been demonstrated in many studies (68–70), pan-cancer analysis of the MYC-PVT1 dysregulation in different cancers yielded a strong correlation between PVT1 and MYC in tumor patients lacking 8q24 locus amplification. An example of PVT1-MYC upregulation without 8q24 amplification was demonstrated to be the result of PVT1 promoter hypomethylation in renal cell cancer and not extra copies of the genes as seen in mammary glands tumors (71). The lower the methylation levels of PVT1 promoter, the higher the levels of the PVT1 and MYC expression and the worse the prognosis of these patients.

PVT1 and c-myc interaction may also be fostered by transcriptional factors that activate the expression of either gene. Two binding sites for FOXM1 (a transcriptional factor that promotes oncogenesis) was found on the promoter region of PVT1 in gastric cancer cells (48). The effect of FOXM1 on PVT1 transcription was also observed on c-myc protein levels. A combination of an overexpression of PVT1 or FOXM1 led to a corresponding elevation in c-myc protein level resulting in oncogenesis. PVT1 exon 1 (the closest to MYC) was reported to have the highest correlation of expression in proximity to MYC when compared to PVT1 exon 4 and exon 8 in CRC (72). The expression of c-myc control genes including FUBP1, NPM1, and EZH2 also correlated positively with the expression of PVT1 exons 1, 4, and 8 in CRC patients. This collaboration suggests an indirect regulation of PVT1 through the regulators of c-myc transcription.

Rearrangements of MYC stemming from microhomology-mediated end joining were located by sequencing data in 36% of patients newly diagnosed with multiple myeloma and resulted in increased expression of c-myc and PVT1 (73). In the 8q24 region, the scramble of the Topologically Associated Domain (TAD) as a result of translocation breakpoints within led to its rearrangements. The new domain formed by the interaction of the super enhancer from the partner loci adjacent to MYC gave rise to an overexpression of MYC.

A promoter competition model (as described by Cho et al.) (74), proposed to unravel the puzzle of c-myc and PVT1 interactions indicated that the PVT1 promoter regulates MYC's transcription in breast cancer as a result of the PVT1 promoter (not PVT1 RNA) acting as a DNA boundary element to regulate the transcription of MYC. Using CRISPRi-PVT1 to silence PVT1 transcription resulted in increased MYC expression in most of the cells in the experiment. The promoters of PVT1 and MYC compete for interactions with the four intragenic enhancers in the PVT1 locus to allow regulation. Usually, these four enhancers preferentially contact PVT1 promoter over the locus of MYC. Silencing PVT1 effectuates the enhancers to contact MYC promoter instead. This contrasts with the action of PVT1 RNA prolonging MYC protein level. Interestingly, CRISPRi-MYC promoter also increased PVT1 transcription.

This model might explain the tissue-specificity of PVT1 expression based on some outcomes in this study. The CRISPRi-PVT1 did not induce an increase in MYC transcription in some cell types, and not all cancers examined had mutations of the PVT1 promoter. Alternatively, PVT1 contribution to tumorigenesis may be independent of MYC (14, 75). This suggests different mechanisms of PVT1 and MYC cooperation in different cancers.

## PVT1 Gene Fusions

PVT1 fusion genes were first described when amplified allele of MYC in colorectal adenocarcinoma cell line showed displacement of Exon 1 and Intron 1 of MYC by PVT1 resulting in DNA rearrangement (63). In combination with coamplification of MYC and PVT1, this DNA rearrangement/fusion genes resulted in the transcription of chimeric RNA transcripts. The PVT1 domain is also noted for frequent translocations in mouse plasmacytomas (76, 77) It was presumed that the rearrangements occurred before the amplification observed.

In the Medulloblastoma Advanced Genomics International Consortium (MAGIC) study, RNA-seq of medulloblastoma Group 3 tumors identified two different PVT1 gene fusions (78). Both types involved the 5′ end of PVT1 (with exon 1 or exon 1 and 3) fused to MYC (exons 2 and 3). Whole Genome sequencing carried out on these PVT1 fusion tumors further revealed genomic rearrangements on chr8q and deletion of chr17p. PVT1 was also shown to be linked with NDRG1, thereby forming the gene fusion PVT1-NDRG1. Computational biology analysis suggested chromothripsis as the process responsible for the creation these gene fusion transcripts. Additionally, RT-PCR demonstrated the upregulation of miR-1204 in PVT1-MYC fusion (+) Group 3 tumors and an increase in cell proliferation of specific medulloblastoma cells; proposing a regulatory system among fusion partners in oncogenesis.

The episome model (79), rather than the chromothripsis process was suggested as the major mechanism for chimeric transcripts generated from the 8q24-amplified genes in AML (80). 5′PVT1/3′CCDC26 chimera and the reciprocal 5′NSMCE2/ 3′PVT1 were detected by WGS and RNA-seq in 23 AML cell lines. Using ChimeraScan and FusionMap, these chimeric transcripts were shown to be closely connected with unusual gene expression profile at areas of amplification and breakpoints.

## PVT1 in Prostate Cancer

The second leading cause of cancer death in men in the United States is prostate cancer; which is also most common cancer among men, except for skin cancer^1^. About 1 man in 9 will be diagnosed with prostate cancer during his lifetime^2^. Data from many sources suggest that PVT1 is a major contributor to the development of prostate cancer (81, 82). A CCK-8 (Cell Counting Kit-8) assay assessment of the effect of PVT1 downregulation in PC3 and DU145 cells showed an inhibition of cell proliferation (83). Confirming this effect in the same study, PVT1 downregulation also reduced the size of tumors in mice. Admixture mapping/scanning has identified multiple regions within 8q24 as a prostate cancer risk locus in African-American men (84, 85). An increased expression of PVT1 was observed when the risk allele of rs378854 at chromosome 8q24 is present in prostate tissue, thereby increasing the risk for prostate cancer (86).

Studies carried out have shown that the mechanisms underlying the regulation of PVT1 in prostate cancer vary considerably. Methylation of the miR-146a promoter induced by the overexpressed PVT1 resulted in the down-regulation of miR-146a and upregulation of PVT1 in prostate cancer (87). This negative regulation of miRNAs is seen in many cancers where PVT1 is overexpressed (22, 27). Consistent with this pattern, PVT1 negatively regulates and reduces the expression of miR-186 as ceRNA in prostate cancer (88). This study further shows that when siRNA-PVT1 or pcDNA-PVT1 was co-transfected with a miR-186 inhibitor or miR186 mimics, Twist1 (a transcription factor associated with EMT) was downregulated by silencing PVT1. On the other hand, co-transfection of siRNA-PVT1 and miR-186 resulted in the upregulation of Twist1.

MiRNA-1207-3p, encoded by PVT1 is significantly under expressed in prostate cancer cell lines whereas apoptosis is induced in prostate cancer by the overexpression of this microRNA (89). The adhesion molecule CD44 was identified as one of the factors under negative regulation by miR-34a in prostate cancer cells (90). An increased expression of miR-34a in CD44+ prostate cancer cells prohibited prostate cancer metastasis. These findings indicate that the mechanisms of miRNAs regulation by PVT1 in prostate cancer certainly involves other coactivators and coregulators.

Additionally, the effect of neighboring genes highlights the complexity of PVT1 in prostate cancer. PVT1 knockdown significantly accelerated prostate cancer cells apoptosis and downregulated the expression of c-myc in an *in vitro* study (56). Both PVT1 and MYC have been suggested as co-regulators of each (27, 47, 48). A molecular assessment of SNP-SNP interactions in the CASC11-MYC-PVT1 region (CASC11 is also located on 8q24) utilizing SNP interaction pattern identifier (SIPI) revealed the CASC11-PVT1 interaction as the most common gene-gene interaction (55 out of 79 pairs) amid 79 top SNP pairs (91). This interaction involving rs16902359 in CASC11 and PVT1 plays a great role in SNP-SNP interactions and could be linked with the high risk of prostate cancer in men of African Ancestry (MoAA). The highest incidence rates for prostate cancer are among (MoAA) men, who have a higher risk of prostate cancer than white American men (92).

**Figure 3:**
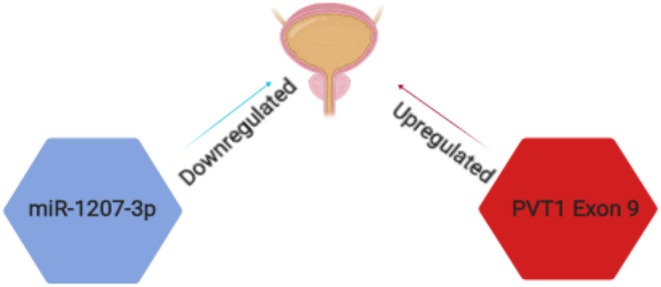
Schematic representation of PVT1 transcripts implicated in prostate cancer. PVT1 exon 9 (red) is significantly overexpressed while miR-1207-3p (blue) is significantly under expressed in prostate cancer in men of African Ancestry.

Although many studies have been carried out on the role of PVT1 in cancer, the specific roles of PVT1 transcripts in prostate cancer have been examined by very few groups. For example, the Ogunwobi laboratory has found that PVT1 exon 9 is a major regulatory component of the PVT1 gene in RWPE1 (human prostate epithelial cell line) (93). The expressions of PVT1 exons 4A, 4B and c-MYC were decreased upon silencing PVT1 exon 9 in RWPE1 cells. Knock down of PVT1 exons 4A and 4B in turn did not yield similar effects. The Ogunwobi laboratory also reported that PVT1 exon 9 is significantly overexpressed in prostate cancer cell lines derived from men of African Ancestry (MoAA) (94) (Figure 3). This observation was made from comparing the relative expression of PVT1 exons in different cell lines derived from men of different ethnic and racial backgrounds. In a more recent study by the Ogunwobi laboratory, PVT1 exon 9 was found to be upregulated in prostate cancer tissues when compared to normal prostate tissues (95). This is consistent with previous studies on the expression of PVT1 exon 9 in prostate cancer cell lines (96). PVT1 exon 9 overexpression in RWPE1 cells promoted cell proliferation and migration compared with the non-transfected cells. A possible mechanism for the oncogenic function of PVT1 exon 9 is by the regulation of PCNA (Proliferating Cell Nuclear Antigen) expression in prostate cancer. In the same study, western blotting performed showed an overexpression of PCNA in cell lysates of RWPE1 cells transfected with PVT1 exon 9 compared to the non-transfected cells. An *in vivo* experiment confirmed the tumor initiation and tumor progression capability of PVT1 exon 9 (97). Male mice implanted with RWPE1 cells overexpressing PVT1 exon 9 developed malignant tumors. Histopathologic investigation of the tumors revealed features typical of the neuroendocrine subtype of aggressive prostate cancer. PVT1 exon 9 was also shown to confer resistance to androgen deprivation therapy in prostate cancer cells overexpressing PVT1 exon 9. These insights into the mechanisms of aggressive prostate cancer deserve further study.

Furthermore, the Ogunwobi laboratory has also described correlation between miR-1207-3p expression and clinicopathological features of prostate cancer [(66); Figure 3]. A decreased expression of miR-1207-3p was found in prostate cancer tissue samples of patients who succumbed to prostate cancer specific death. The expression of miR-1207-3p in prostate cancer tissues of MoAA (men of African Ancestry) was significantly decreased and might explain the genetic differences underlying racial disparity in prostate cancer. Although these studies have opened the possibilities of new diagnostic biomarkers in prostate cancer, further clarification are needed to explore how these transcripts are regulated and if they regulate each other.

## Therapeutic Implications

Targeting PVT1 in cancer is a promising novel approach to cancer treatment. Many of PVT1's interacting factors (including MYC) with PVT1 in carcinogenesis are essential transcription factors that have been very difficult to drug. Modulating the functions of PVT1 where implicated in cancer may give rise to successful anti-cancer therapies. Modulation of PVT1 can take many forms including potential development of specific PVT1 inhibitors. Some modulation of IncRNA functions have been established with future anticancer potentials (96).

The role of PVT1 as a cancer biomarker is progressively becoming established. It has been demonstrated that PVT1 is quantifiable in cancer cells and in serum from cancer patients (97–99). Potentially, PVT1 can be used in early diagnosis of cancer or for monitoring response to cancer treatment, and chemoresistance. The participation of PVT1 in many signaling pathways and the tissue-specific mechanisms of action suggests the need to tailor any PVT1-based therapeutic strategies developed.

## Conclusion

**Figure 4:**
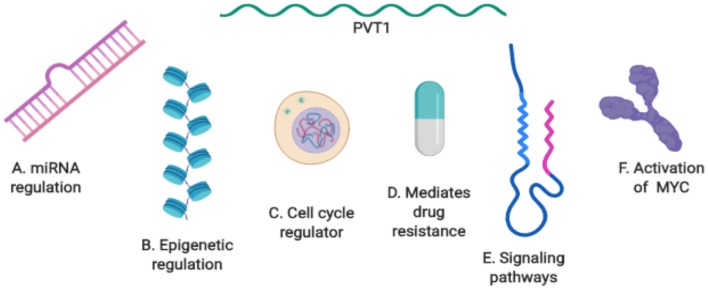
Summary of PVT1 mechanisms of action in cancer. **(A)** PVT1 regulation by miRNA. **(B)** Epigenetic regulation by PVT1. **(C)** PVT1: a regulator of the cell cycle. **(D)** PVT1 mediates drug resistance. **(E)** PVT1 at the center of biological axes and signaling pathways. **(F)** The activation of PVT1 and MYC in cancer.

Despite the remarkable progress already made in uncovering the role of PVT1 and its mechanisms of action in cancer (Figure 4), there is so much more about PVT1 that remains unknown. Details of PVT1's fundamental biology, such as its biogenesis, transcription, alternative splicing, etc. remain unknown. Furthermore, a much more comprehensive understanding of PVT1 specific interactions with downstream molecular mediators in different cancers is still needed to be able to fully exploit PVT1 for clinical applications. How and when to target PVT1 in cancer remains an important focus. Further studies are needed to explore how PVT1 transcripts are regulated by each other, and which PVT1 transcripts participate in regulatory activities with neighboring genes, as well as the functions of PVT1 transcripts in different types of cancers.

## Author Contributions

OTO and OOO conceived of this work. OTO completed first draft of the manuscript. OOO completed final version of the manuscript and supervised all of the work. CO and GP participated in parts of the first draft of the manuscript. GP participated in creating figures for the manuscript. All authors contributed intellectually to manuscript content.

### Conflict of Interest

OOO is a Co-Founder of NucleoBio, Inc., a City University of New York start-up biotechnology company. The remaining authors declare that the research was conducted in the absence of any commercial or financial relationships that could be construed as a potential conflict of interest. The handling editor is currently editing co-organizing a Research Topic with one of the authors OOO, and confirms the absence of any other collaboration.

## Footnotes

^1^https://www.cancer.net/cancer-types/prostate-cancer/statistics

^2^https://www.cancer.org/cancer/prostate-cancer/about/key-statistics.html

## References

[1] Kapranov P Cheng J Dike S Nix DA Duttagupta R Willingham AT. RNA maps reveal new RNA classes and a possible function for pervasive transcription. Science. (2007) 316:1484–8. 10.1126/science.1138341 17510325

[2] Wang KC Chang HY. Molecular mechanisms of long noncoding RNAs. Mol Cell. (2011) 43:904–14. 10.1016/j.molcel.2011.08.018 21925379 PMC3199020

[3] ENCODE Project Consortium Identification and analysis of functional elements in 1% of the human genome by the ENCODE pilot project. Nature. (2007) 447:799 10.1038/nature05874 17571346 PMC2212820

[4] Mattick JS. The genetic signatures of noncoding RNAs. PLoS Genet. (2009) 5:e1000459. 10.1371/journal.pgen.1000459 19390609 PMC2667263

[5] van Bakel H Nislow C Blencowe BJ Hughes TR. Most dark matter transcripts are associated with known genes. PLoS Biol. (2010) 8:e1000371. 10.1371/journal.pbio.1000371 20502517 PMC2872640

[6] Wang X Arai S Song X Reichart D Du K Pascual G. Induced ncRNAs allosterically modify RNA-binding proteins *in cis* to inhibit transcription. Nature. (2008) 454:126. 10.1038/nature06992 18509338 PMC2823488

[7] Schwartz JC Younger ST Nguyen NB Hardy DB Monia BP Corey DR. Antisense transcripts are targets for activating small RNAs. Nat Struct Mol Biol. (2008) 15:842. 10.1038/nsmb.1444 18604220 PMC2574822

[8] Kino T Hurt DE Ichijo T Nader N Chrousos GP. Noncoding RNA gas5 is a growth arrest–and starvation-associated repressor of the glucocorticoid receptor. Sci Signal. (2010) 3:ra8. 10.1126/scisignal.2000568 20124551 PMC2819218

[9] Rinn JL Kertesz M Wang JK Squazzo SL Xu X Brugmann SA. Functional demarcation of active and silent chromatin domains in human HOX loci by noncoding RNAs. Cell. (2007) 129:1311–23. 10.1016/j.cell.2007.05.022 17604720 PMC2084369

[10] Prensner JR Iyer MK Balbin OA Dhanasekaran SM Cao Q Brenner JC. Transcriptome sequencing across a prostate cancer cohort identifies PCAT-1, an unannotated lincRNA implicated in disease progression. Nat Biotechnol. (2011) 29:742. 10.1038/nbt.1914 21804560 PMC3152676

[11] Gupta RA Shah N Wang KC Kim J Horlings HM Wong DJ. Long non-coding RNA HOTAIR reprograms chromatin state to promote cancer metastasis. Nature. (2010) 464:1071. 10.1038/nature08975 20393566 PMC3049919

[12] Shtivelman E Bishop JM. Effects of translocations on transcription from PVT. Mol Cell Biol. (1990) 10:1835–9. 10.1128/MCB.10.4.1835 2181290 PMC362296

[13] Huppi K David S. Chimeric transcripts with an open reading frame are generated as a result of translocation to the Pvt-1 region in mouse B-cell tumors. Int J Cancer. (1994) 59:848–51. 10.1002/ijc.2910590623 7989128

[14] Guan Y Kuo WL Stilwell JL Takano H Lapuk AV Fridlyand J. Amplification of PVT1 contributes to the pathophysiology of ovarian and breast cancer. Clin Cancer Res. (2007) 13:5745–55. 10.1158/1078-0432.CCR-06-2882 17908964

[15] Carramusa L Contino F Ferro A Minafra L Perconti G Giallongo A. The PVT-1 oncogene is a Myc protein target that is overexpressed in transformed cells. J Cell Physiol. (2007) 213:511–8. 10.1002/jcp.21133 17503467

[16] Liu E Liu Z Zhou Y Mi R Wang D. Overexpression of long non-coding RNA PVT1 in ovarian cancer cells promotes cisplatin resistance by regulating apoptotic pathways. Int J Clin Exp Med. (2015) 8:20565. 26884974 PMC4723819

[17] Paci P Teresa C Lorenzo F. Computational analysis identifies a sponge interaction network between long non-coding RNAs and messenger RNAs in human breast cancer. BMC Syst Biol. (2014) 8:83. 10.1186/1752-0509-8-83 25033876 PMC4113672

[18] Wang F Yuan JH Wang SB Yang F Yuan SX Ye C. Oncofetal long noncoding RNA PVT1 promotes proliferation and stem cell-like property of hepatocellular carcinoma cells by stabilizing NOP2. Hepatology. (2014) 60:1278–90. 10.1002/hep.27239 25043274

[19] Tseng YY Moriarity BS Gong W Akiyama R Tiwari A Kawakami H. PVT1 dependence in cancer with MYC copy-number increase. Nature. (2014) 512:82. 10.1038/nature13311 25043044 PMC4767149

[20] Kong R Zhang EB Yin DD You LH Xu TP Chen WM. Long noncoding RNA PVT1 indicates a poor prognosis of gastric cancer and promotes cell proliferation through epigenetically regulating p15 and p16. Mol Cancer. (2015) 14:82. 10.1186/s12943-015-0355-8 25890171 PMC4399399

[21] Zhou Q Chen J Feng J Wang J. Long noncoding RNA PVT1 modulates thyroid cancer cell proliferation by recruiting EZH2 and regulating thyroid-stimulating hormone receptor (TSHR). Tumor Biol. (2016) 37:3105–13. 10.1007/s13277-015-4149-9 26427660

[22] Wang D Hu Y. Long non-coding RNA PVT1 competitively binds microRNA-424-5p to regulate CARM1 in radiosensitivity of non-small-cell lung cancer. Mol Ther Nucleic Acids. (2019) 16:130–40. 10.1016/j.omtn.2018.12.006 30861415 PMC6411630

[23] Shang AQ Wang WW Yang YB Gu CZ Ji P Chen C Knockdown of long non-coding RNA PVT1 suppresses cell proliferation and invasion of colorectal cancer via upregulation of microRNA-214-3p. Am J Physiol Gastrointest Liver Physiol. (2019) 317:G222–32. 10.1152/ajpgi.00357.2018 31125260

[24] Alessio E Buson L Chemello F Peggion C Grespi F Martini P. Single cell analysis reveals the involvement of the long non-coding RNA Pvt1 in the modulation of muscle atrophy and mitochondrial network. Nucleic Acids Res. (2019) 47:1653–70. 10.1093/nar/gkz007 30649422 PMC6393313

[25] Huang T Liu HW Chen JQ Wang SH Hao LQ Liu M. The long noncoding RNA PVT1 functions as a competing endogenous RNA by sponging miR-186 in gastric cancer. Biomed Pharmacother. (2017) 88:302–8. 10.1016/j.biopha.2017.01.049 28122299

[26] Lan T Yan X Li Z Xu X Mao Q Ma W. Long non-coding RNA PVT1 serves as a competing endogenous RNA for miR-186-5p to promote the tumorigenesis and metastasis of hepatocellular carcinoma. Tumor Biol. (2017) 39:1010428317705338. 10.1177/1010428317705338 28656879

[27] Zhou Q Chen F Zhao J Li B Liang Y Pan W. Long non-coding RNA PVT1 promotes osteosarcoma development by acting as a molecular sponge to regulate miR-195. Oncotarget. (2016) 7:82620. 10.18632/oncotarget.13012 27813492 PMC5347719

[28] Zhang S Guanli Z Jingying L. Long noncoding RNA PVT1 promotes cervical cancer progression through epigenetically silencing miR-200b. APMIS. (2016) 124:649–58. 10.1111/apm.12555 27272214

[29] Shen C-J Cheng YM Wang C-L. LncRNA PVT1 epigenetically silences miR-195 and modulates EMT and chemoresistance in cervical cancer cells. J Drug Target. (2017) 25:637–44. 10.1080/1061186X.2017.1307379 28296507

[30] Tang J Li Y Sang Y Yu B Lv D Zhang W. LncRNA PVT1 regulates triple-negative breast cancer through KLF5/beta-catenin signaling. Oncogene. (2018) 37:4723. 10.1038/s41388-018-0310-4 29760406

[31] Yu X Jianpei Z Yaguang H. Long non-coding RNA PVT1 functions as an oncogene in human colon cancer through miR-30d-5p/RUNX2 axis. J BUON. (2018) 23:48–54. 29552759

[32] Fu C Li D Zhang X Liu N Chi G Jin X. LncRNA PVT1 facilitates tumorigenesis and progression of glioma via regulation of MiR-128-3p/GREM1 Axis and BMP signaling pathway. Neurotherapeutics. (2018) 15:1139–57. 10.1007/s13311-018-0649-9 30120709 PMC6277294

[33] Wu BQ Jiang Y Zhu F Sun DL He XZ Long noncoding RNA PVT1 promotes EMT and cell proliferation and migration through downregulating p21 in pancreatic cancer cells. Technol Cancer Res Treat. (2017) 16:819–27. 10.1177/1533034617700559 PMC5762037 28355965

[34] Salehi M Mohammadreza S Marzieh B. Knockdown of long noncoding RNA plasmacytoma variant translocation 1 with antisense locked nucleic acid GapmeRs exerts tumor-suppressive functions in human acute erythroleukemia cells through downregulation of C-MYC expression. Cancer Biother Radiopharm. (2018) 34:371–9. 10.1089/cbr.2018.2510 30141968

[35] Bartel DP. MicroRNAs: genomics, biogenesis, mechanism, and function. Cell. (2004) 116:281–97. 10.1016/S0092-8674(04)00045-5 14744438

[36] Denli AM Tops BB Plasterk RH Ketting RF Hannon GJ. Processing of primary microRNAs by the Microprocessor complex. Nature. (2004) 432:231. 10.1038/nature03049 15531879

[37] Huppi K Volfovsky N Runfola T Jones TL Mackiewicz M Martin SE. The identification of microRNAs in a genomically unstable region of human chromosome 8q24. Mol Cancer Res. (2008) 6:212–21. 10.1158/1541-7786.MCR-07-0105 18314482

[38] Barsotti AM Beckerman R Laptenko O Huppi K Caplen NJ Prives C. p53-Dependent induction of PVT1 and miR-1204. J Biol Chem. (2012) 287:2509–19. 10.1074/jbc.M111.322875 22110125 PMC3268411

[39] Li T Meng X Yang W. Long noncoding RNA PVT1 acts as a Sponge to inhibit microRNA-152 in gastric cancer cells. Dig Dis Sci. (2017) 62:3021–8. 10.1007/s10620-017-4508-z 28258379

[40] Li PD Hu JL Ma C Ma H Yao J Chen LL. Upregulation of the long non-coding RNA PVT1 promotes esophageal squamous cell carcinoma progression by acting as a molecular sponge of miR-203 and LASP1. Oncotarget. (2017) 8:34164. 10.18632/oncotarget.15878 28404954 PMC5470958

[41] Wu D Li Y Zhang H Hu X. Knockdown of Lncrna PVT1 enhances radiosensitivity in non-small cell lung cancer by sponging Mir-195. Cell Physiol Biochem. (2017) 42:2453–66. 10.1159/000480209 28848163

[42] Zhao L Kong H Sun H Chen Z Chen B Zhou M. LncRNA-PVT1 promotes pancreatic cancer cells proliferation and migration through acting as a molecular sponge to regulate miR-448. J Cell Physiol. (2018) 233:4044–55. 10.1002/jcp.26072 28657147

[43] Xue W Chen J Liu X Gong W Zheng J Guo X. PVT1 regulates the malignant behaviors of human glioma cells by targeting miR-190a-5p and miR-488-3p. Biochim Biophys Acta Mol Basis Dis. (2018) 1864:1783–94. 10.1016/j.bbadis.2018.02.022 29501773

[44] Song J Wu X Liu F Li M Sun Y Wang Y. Long non-coding RNA PVT1 promotes glycolysis and tumor progression by regulating miR-497/HK2 axis in osteosarcoma. Biochem Biophys Res Commun. (2017) 490:217–24. 10.1016/j.bbrc.2017.06.024 28602700

[45] Buczkowicz P Hoeman C Rakopoulos P Pajovic S Letourneau L Dzamba M. Genomic analysis of diffuse intrinsic pontine gliomas identifies three molecular subgroups and recurrent activating ACVR1 mutations. Nat Genet. (2014) 46:451. 10.1038/ng.2936 24705254 PMC3997489

[46] Wan L Sun M Liu GJ Wei CC Zhang EB Kong R. Long noncoding RNA PVT1 promotes non–small cell lung cancer cell proliferation through epigenetically regulating LATS2 expression. Mol Cancer Ther. (2016) 15:1082–94. 10.1158/1535-7163.MCT-15-0707 26908628

[47] Yang A Handong W Xiaobing Y. Long non-coding RNA PVT1 indicates a poor prognosis of glioma and promotes cell proliferation and invasion via target EZH2. Biosci Rep. (2017) 37:BSR20170871. 10.1042/BSR20170871 29046366 PMC6435466

[48] Xu MD Wang Y Weng W Wei P Qi P Zhang Q. A positive feedback loop of lncRNA-PVT1 and FOXM1 facilitates gastric cancer growth and invasion. Clin Cancer Res. (2017) 23:2071–80. 10.1158/1078-0432.CCR-16-0742 27756785

[49] Yan C Chen Y Kong W Fu L Liu Y Yao Q PVT 1-derived miR-1207-5p promotes breast cancer cell growth by targeting STAT 6. Cancer Sci. (2017) 108:868–76. 10.1111/cas.13212 28235236 PMC5448618

[50] Chen L Ma D Li Y Li X Zhao L Zhang J. Effect of long non-coding RNA PVT1 on cell proliferation and migration in melanoma. Int J Mol Med. (2018) 41:1275–82. 10.3892/ijmm.2017.3335 29286144 PMC5819911

[51] Abbas T Anindya D. p21 in cancer: intricate networks and multiple activities. Nat Rev Cancer. (2009) 9:400. 10.1038/nrc2657 19440234 PMC2722839

[52] Li X Chen W Wang H Wei Q Ding X Li W. Amplification and the clinical significance of circulating cell-free DNA of PVT1 in breast cancer. Oncol Rep. (2017) 38:465–71. 10.3892/or.2017.5650 28534994

[53] Zhang XW Bu P Liu L Zhang XZ Li J. Overexpression of long non-coding RNA PVT1 in gastric cancer cells promotes the development of multidrug resistance. Biochem Biophys Res Commun. (2015) 462:227–32. 10.1016/j.bbrc.2015.04.121 25956062

[54] Bao B Ali S Banerjee S Wang Z Logna F Azmi AS. Curcumin analogue CDF inhibits pancreatic tumor growth by switching on suppressor microRNAs and attenuating EZH2 expression. Cancer Res. (2012) 72:335–45. 10.1158/0008-5472.CAN-11-2182 22108826 PMC3792589

[55] Yoshida K Toden S Ravindranathan P Han H Goel A. Curcumin sensitizes pancreatic cancer cells to gemcitabine by attenuating PRC2 subunit EZH2, and the lncRNA PVT1 expression. Carcinogenesis. (2017) 38:1036–46. 10.1093/carcin/bgx065 29048549 PMC5862331

[56] He Y Jing Y Wei F Tang Y Yang L Luo J. Long non-coding RNA PVT1 predicts poor prognosis and induces radioresistance by regulating DNA repair and cell apoptosis in nasopharyngeal carcinoma. Cell Death Dis. (2018) 9:235. 10.1038/s41419-018-0265-y 29445147 PMC5833381

[57] Bakkus MH Brakel-van KP Michiels JJ Benner R. Amplification of the c-myc and the pvt-like region in human multiple myeloma. Oncogene. (1990) 5:1359–64. 2216459

[58] Zhao J Du P Cui P Qin Y Wu J Zhou Z. LncRNA PVT1 promotes angiogenesis via activating the STAT3/VEGFA axis in gastric cancer. Oncogene. (2018) 37:4094. 10.1038/s41388-018-0250-z 29706652

[59] Zhang X Feng W Zhang J Ge L Zhang Y Jiang X Long non-coding RNA PVT1 promotes epithelial-mesenchymal transition via the TGF-β/Smad pathway in pancreatic cancer cells. Oncol Rep. (2018) 40:1093–102. 10.3892/or.2018.6462 29845201

[60] Cui M Chang Y Fang QG Du W Wu JF Wang JH. Non-coding RNA Pvt1 promotes cancer stem cell–like traits in nasopharyngeal cancer via inhibiting miR-1207. Pathol Oncol Res. (2019) 25:1411–22. 10.1007/s12253-018-0453-1 30141114

[61] Li W Zheng Z Chen H Cai Y Xie W. Knockdown of long non-coding RNA PVT1 induces apoptosis and cell cycle arrest in clear cell renal cell carcinoma through the epidermal growth factor receptor pathway. Oncol Lett. (2018) 15:7855–63. 10.3892/ol.2018.8315 29725475 PMC5920359

[62] Graham M Jerry MA. Chromosome 8 breakpoint far 3'of the c-myc oncogene in a Burkitt's lymphoma 2; 8 variant translocation is equivalent to the murine pvt-1 locus. EMBO J. (1986) 5:2845. 10.1002/j.1460-2075.1986.tb04578.x 3024964 PMC1167233

[63] Shtivelman E Bishop JM. The PVT gene frequently amplifies with MYC in tumor cells. Mol Cell Biol. (1989) 9:1148–54. 10.1128/MCB.9.3.1148 2725491 PMC362705

[64] Rao PH Zhao S Zhao YJ Yu A Rainusso N Trucco M Coamplification of M yc/P vt1 and homozygous deletion of N lrp1 locus are frequent genetics changes in mouse osteosarcoma. Genes Chromosomes Cancer. (2015) 54:796–808. 10.1002/gcc.22291 26355645

[65] Yazdi N Houshmand M Atashi A Kazemi A Najmedini AA Zarif MN. Long noncoding RNA PVT1: potential oncogene in the development of acute lymphoblastic leukemia. Turk J Biol. (2018) 42:405–13. 10.3906/biy-1801-46 30930624 PMC6438125

[66] You L Wang H Yang G Zhao F Zhang J Liu Z Gemcitabine exhibits a suppressive effect on pancreatic cancer cell growth by regulating processing of PVT 1 to miR1207. Mol Oncol. (2018) 12:2147–64. 10.1002/1878-0261.12393 30341811 PMC6275279

[67] Sarver AL Murray CD Temiz NA Tseng YY Bagchi A. MYC and PVT1 synergize to regulate RSPO1 levels in breast cancer. Cell Cycle. (2016) 15:881–5. 10.1080/15384101.2016.1149660 26889781 PMC4889295

[68] Raeder MB Birkeland E Trovik J Krakstad C Shehata S Schumacher S. Integrated genomic analysis of the 8q24 amplification in endometrial cancers identifies ATAD2 as essential to MYC-dependent cancers. PLoS ONE. (2013) 8:e54873. 10.1371/journal.pone.0054873 23393560 PMC3564856

[69] Fromont G Godet J Peyret A Irani J Celhay O Rozet F. 8q24 amplification is associated with Myc expression and prostate cancer progression and is an independent predictor of recurrence after radical prostatectomy. Hum Pathol. (2013) 44:1617–23. 10.1016/j.humpath.2013.01.012 23574779

[70] Anauate AC Leal MF Wisnieski F Santos LC Gigek CO Chen ES. Analysis of 8q24. 21 miRNA cluster expression and copy number variation in gastric cancer. Fut Med Chem. (2019) 11:947–58. 10.4155/fmc-2018-0477 31141411

[71] Posa I Carvalho S Tavares J Grosso AR. A pan-cancer analysis of MYC-PVT1 reveals CNV-unmediated deregulation and poor prognosis in renal carcinoma. Oncotarget. (2016) 7:47033. 10.18632/oncotarget.9487 27366943 PMC5216921

[72] Guo K Yao J Yu Q Li Z Huang H Cheng J. The expression pattern of long non-coding RNA PVT1 in tumor tissues and in extracellular vesicles of colorectal cancer correlates with cancer progression. Tumor Biol. (2017) 39:1010428317699122. 10.1177/1010428317699122 28381186

[73] Mikulasova A Ashby C Tytarenko RG Qu P Rosenthal A Dent JA Microhomology-mediated end joining drives complex rearrangements and over expression of MYC and PVT1 in multiple myeloma. Haematologica. (2019) 2019:217927 10.3324/haematol.2019.217927 PMC7109748 31221783

[74] Cho SW Xu J Sun R Mumbach MR Carter AC Chen YG. Promoter of lncRNA gene PVT1 is a tumor-suppressor DNA boundary element. Cell. (2018) 173:1398–412. 10.1016/j.cell.2018.03.068 29731168 PMC5984165

[75] Takahashi Y Sawada G Kurashige J Uchi R Matsumura T Ueo H. Amplification of PVT-1 is involved in poor prognosis via apoptosis inhibition in colorectal cancers. Br J Cancer. (2014) 110:164. 10.1038/bjc.2013.698 24196785 PMC3887297

[76] Cory S Graham M Webb E Corcoran L Adams JM. Variant (6; 15) translocations in murine plasmacytomas involve a chromosome 15 locus at least 72 kb from the c-myc oncogene. EMBO J. (1985) 4:675–81. 10.1002/j.1460-2075.1985.tb03682.x 3924592 PMC554241

[77] Webb E Adams JM Cory S. Variant (6; 15) translocation in a murine plasmacytoma occurs near an immunoglobulin κ gene but far from the myc oncogene. Nature. (1984) 312:777. 10.1038/312777a0 6440031

[78] Northcott PA Shih DJ Peacock J Garzia L Morrissy AS Zichner T. Subgroup-specific structural variation across 1,000 medulloblastoma genomes. Nature. (2012) 488:49. 10.1038/nature11327 22832581 PMC3683624

[79] Storlazzi CT Lonoce A Guastadisegni MC Trombetta D D'Addabbo P Daniele G. Gene amplification as double minutes or homogeneously staining regions in solid tumors: origin and structure. Genome Res. (2010) 20:1198–206. 10.1101/gr.106252.110 20631050 PMC2928498

[80] L'Abbate A Tolomeo D Cifola I Severgnini M Turchiano A Augello B. Correction: MYC-containing amplicons in acute myeloid leukemia: genomic structures, evolution, and transcriptional consequences. Leukemia. (2018) 32:2304. 10.1038/s41375-018-0177-y 29985446 PMC7608236

[81] Soubra A Badrinath K Anindya B Mp61-06 increased pvt1 expression correlates with advanced stage and hormone resistance of prostate cancer. J Urol. (2015) 4:e748–9. 10.1016/j.juro.2015.02.2187

[82] Yang J Li C Mudd A Gu X. LncRNA PVT1 predicts prognosis and regulates tumor growth in prostate cancer. Biosci Biotechnol Biochem. (2017) 81:2301–6. 10.1080/09168451.2017.1387048 29050519

[83] Wan B Wu HY Lv DJ Zhou XM Zhong LR Lei B. Downregulation of lncRNA PVT1 expression inhibits proliferation and migration by regulating p38 expression in prostate cancer. Oncol Lett. (2018) 16:5160–6. 10.3892/ol.2018.9305 30250582 PMC6144883

[84] Freedman ML Haiman CA Patterson N McDonald GJ Tandon A Waliszewska A. Admixture mapping identifies 8q24 as a prostate cancer risk locus in African-American men. Proc Natl Acad Sci USA. (2006) 103:14068–73. 10.1073/pnas.0605832103 16945910 PMC1599913

[85] Haiman CA Patterson N Freedman ML Myers SR Pike MC Waliszewska A. Multiple regions within 8q24 independently affect risk for prostate cancer. Nat Genet. (2007) 39:638. 10.1038/ng2015 17401364 PMC2638766

[86] Meyer KB Maia AT O'Reilly M Ghoussaini M Prathalingam R Porter-Gill P. A functional variant at a prostate cancer predisposition locus at 8q24 is associated with PVT1 expression. PLoS Genet. (2011) 7:e1002165. 10.1371/journal.pgen.1002165 21814516 PMC3140991

[87] Liu HT Fang L Cheng YX Sun Q. LncRNA PVT1 regulates prostate cancer cell growth by inducing the methylation of miR-146a. Cancer Med. (2016) 5:3512–9. 10.1002/cam4.900 27794184 PMC5224852

[88] Chang Z Jun C Yongsheng S. Long noncoding RNA PVT1 promotes EMT via mediating microRNA-186 targeting of Twist1 in prostate cancer. Gene. (2018) 654:36–42. 10.1016/j.gene.2018.02.036 29452232

[89] Das DK Naidoo M Ilboudo A Park JY Ali T Krampis K. miR-1207-3p regulates the androgen receptor in prostate cancer via FNDC1/fibronectin. Exp Cell Res. (2016) 348:190–200. 10.1016/j.yexcr.2016.09.021 27693493 PMC5077722

[90] Liu C Kelnar K Liu B Chen X Calhoun-Davis T Li H. The microRNA miR-34a inhibits prostate cancer stem cells and metastasis by directly repressing CD44. Nat Med. (2011) 17:211. 10.1038/nm.2284 21240262 PMC3076220

[91] Lin HY Callan CY Fang Z Tung HY Park JY. Interactions of PVT1 and CASC11 on prostate cancer risk in African Americans. Cancer Epidemiol Prev Biomark. (2019) 28:1067–75. 10.1158/1055-9965.EPI-18-1092 30914434 PMC6548667

[92] Bostwick DG Burke HB Djakiew D Euling S Ho SM Landolph J. Human prostate cancer risk factors. Cancer. (2004) 101:2371–490. 10.1002/cncr.20408 15495199

[93] Onagoruwa Onayemi Titilayo (2018). Molecular interactions between PVT1 transcripts and C-Myc. (Diss). New York, NY: Hunter College, Department of Biological Sciences.

[94] Ilboudo A Chouhan J McNeil B Osborne J Ogunwobi O. PVT1 exon 9: a potential biomarker of aggressive prostate cancer? Int J Env Res Public Health. (2016) 13:12. 10.3390/ijerph13010012 26703666 PMC4730403

[95] Pal G Huaman J Levine F Orunmuyi A Olapade-Olaopa EO Onagoruwa OT. Long noncoding RNA from PVT1 Exon 9 is overexpressed in prostate cancer and induces malignant transformation and castration resistance in prostate epithelial cells. Genes. (2019) 10:964. 10.3390/genes10120964 31766781 PMC6969942

[96] Wu T Yantao D. LncRNAs: from basic research to medical application. Int J Biol Sci. (2017) 13:295. 10.7150/ijbs.16968 28367094 PMC5370437

[97] Xie Z Chen X Li J Guo Y Li H Pan X. Salivary HOTAIR and PVT1 as novel biomarkers for early pancreatic cancer. Oncotarget. (2016) 7:25408. 10.18632/oncotarget.8323 27028998 PMC5041913

[98] Cui D Yu CH Liu M Xia QQ Zhang YF Jiang WL. Long non-coding RNA PVT1 as a novel biomarker for diagnosis and prognosis of non-small cell lung cancer. Tumor Biol. (2016) 37:4127–34. 10.1007/s13277-015-4261-x 26490983

[99] Huang C Liu S Wang H Zhang Z Yang Q Gao F. LncRNA PVT1 overexpression is a poor prognostic biomarker and regulates migration and invasion in small cell lung cancer. Am J Transl Res. (2016) 8:5025. 27904703 PMC5126345

